# The Indications for Drainage of the Knee-Joint

**Published:** 1878-06

**Authors:** 


THE INDICATIONS FOR DRAINAGE OF THE KNEE-JOINT.—Dr. J. Scriber, assistant in the Surgical Clinic at Friedburg, recommends drainage of the knee-joint instead of excision, in the following cases: 1. In acute serous inflammation, in the rare event of there being abnormal pain of sufficient severity to affect the patient's general health. 2. In acute prevalent inflammation of the joint, as soon as there is distinct fluctuation; in the rare case of osteomyelitis involving one or both epiphyses; in the prevalent inflammation which may complicate pyæmia, pneumonia, acute infectious diseases and phlegmonous erysipelas of the lower extremities. 3. In chronic serous inflammation of the joint. 4. In fungous granulation (*a*) where the fluid secretion in the joint exceeds the fungous granulation in amount, and where the cartilage is still intact; (*b*) where there is excess of fungous granulation, but where caries is still absent. The presence of caries is a contra-indication for drainage and an indication for excision. The earlier chronic fungous inflammation of a joint comes under treatment, the better hope there is of giving the patient a useful, movable knee-joint by means of drainage.—*Hospital Gazette and Archives of Surgery.*

